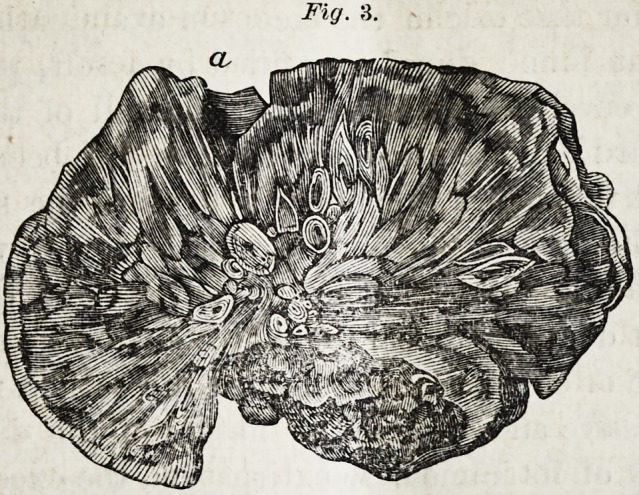# Investigation of a Malformed Tooth of the Lower Jaw

**Published:** 1869-08

**Authors:** O. Salomon


### ARTICLE IV.

### *Investigation of a Malformed Tooth of the Lower Jaw.*

(Translated from the German of Prof. Wedl. By Mr. O. Salomon.)

Dr. Steinberger at a meeting of the Austrian Dentists described an interesting case of a malformed tooth situated in the lower jaw of a young lady aged 18 years, the diagnosis of which was very difficult and highly important.

The history of this case is as follows: The patient presented herself to Dr. S. six months before having an immense swelling on the right side of the lower jaw, extending from near the coronoid process to the second bicuspid, and having a diameter of two inches across the jaw. The enlargment of bone was much greater externally than internally, and the entire tumor on the inside of the cheek was covered with tense mucous membrane. The tumor interfered materially with the closing of the jaw rendering mastication imperfect.

The symptoms manifesting themselves during the eight days prior to her presenting herself, were similar to those from a wisdom tooth which is retarded in its eruption from want of space between the ascending portion of the jaw and the second molar tooth, but in this case no molar teeth were present, the first molar having been removed some years before, and the second molar never having made its appearance.

Dr. Steinberger could find nothing in the lower jaw which would offer an obstacle to the free eruption of the wisdom tooth, but in the upper jaw he found that the superior second molar of the right side, on closing the mouth, pressed upon the top of the tumor in the lower jaw. To prevent such a closure of the jaws a small piece of hard rubber was formed to fit the grinding surfaces of the superior and inferior molars of the left side, which were present, and by this means the jaws were prevented from coming together. Previous to the application of this piece of rubber, intense pain followed the closing of the mouth from the opposing molar tooth pressing upon the tumor; after the application of the rubber, however, no such pain was experienced, and the inflammation resulting in suppuration the pus was let out by an incision with the lancet. The jaw, however, continued enlarged, and three weeks later a small opening made its appearance in the gum, at the point formerly pressed upon by the molar tooth in the opposite jaw. A probe introduced into this opening in the gum revealed the presence of a hard substance, but not, however, so hard as what might be expected from a surface of enamel. The gum was then freely dissected, and a hard yellowish-white formation with an irregular upper surface discovered firmly implanted in the bone of the jaw.

Although this was undoubtedly a tooth formation, yet, there was nothing present to account for the degree of inflammation excited, except it might be its inverted position and its irregular form.

Dr. Steinberger at this stage diagnosed the case as follows: That it was a malformed inverted second molar tooth, the papilla of which in its papillary stage had been disturbed from some unknown cause, and overlapped the papilla of the wisdom tooth, and this latter tooth developing pressed upon the malformed tooth giving rise to a high degree of inflammation.

Owing to the fact that but a small portion of the malformed tooth was visible after the dissection of the gum, it was necessary to remove a considerable portion of the bone of the jaw which covered it before it could be extracted.

Before the operation, however, Dr. Weinlechner was consulted who differed from Dr. Steinberger in his diagnosis of the case, asserting that it was a sarcomatous cyst of the bone. Dr. Steinberger not admitting this view of the case, perforated the tumor with a small drill to the depth of half an inch, but did not find any cyst cavity, nor did any blood escape from the opening made by the drill, or pain to the patient. Still Dr. Weinlechner was not convinced, and Dr. S. detached a small portion from the bony mass and presented it to Dr. Wedl for examination under the microscope.

Dr. Wedl having pronounced the substance to be dentine, it was determined to extract it. Dr. Weinlechner first dissected more of the gum from about the tumor, and by means of an elevator attempted to dislodge the malformed tooth. Failing with this instrument recourse was had to the forceps, but no hold sufficiently strong could be obtained upon it. A screw, known as Serre's screw, was then inserted in the hole before made with the drill, and by means of this Dr. W. succeeded in moving the tooth, but could not dislodge it, and then resorted to the chisel, by means of which he enlarged the opening in the bone sufficient to allow the tooth to pass.

Inserting the edge of the chisel between the bony wall of the cavity and the side of the tumor, a few blows of the mallet dislodged the malformed tooth.

Directly underneath the malformed tooth, in the bottom of the cavity, appeared the partly developed crown of the wisdom tooth.

Prof. Wedl gives the following description of this malformed tooth: In size and form it resembled a chestnut, and its longitudinal diameter measured 29 millimeters, somewhat more than an English inch, and the latitudinal diameter nineteen millimetres; its weight, in a humid condition, being 12.37 gramme, a little more than half an ounce. In color it was yellowish-white, very dense in consistence, its upper surface being convex and covered with many small resistent glandulae.

*Fig.* 1.

(Fig. 1. view of the upper surface of malformed tooth, natural size.) On this surface a small flattened spot appeared, caused by the pressure of the superior molar. The two lateral surfaces were more convex and traversed by canals. On the external margin of the base was a cavity quadilateral in shape, and of a depth equal to between three and four millimeters, corresponding in size and form with the grinding surface of a molar tooth, with two smooth walls, one of which inclined inward, and two irregular ones which inclined outward.

*Fig* 2.

(Fig. 2. view of the base of the tooth, (*a*) internal surface, (*b*) external surface, natural size.) About the periphery of the base, with the exception of that portion occupied by the cavity just referred to, hung a tough cellular fringe having a strong adhesion.

*Fig.* 3.

(Fig 3 represents a vertical section from the malformed tooth twice the natural size, (*a*,)being the cavity formed by the removal of the small piece first subjected to the microscope. The greater part of the mass was composed of dentine; on the base was found enamel, and on the upper convex portion cementum.

## Figures and Tables

**Fig. 1. f1:**
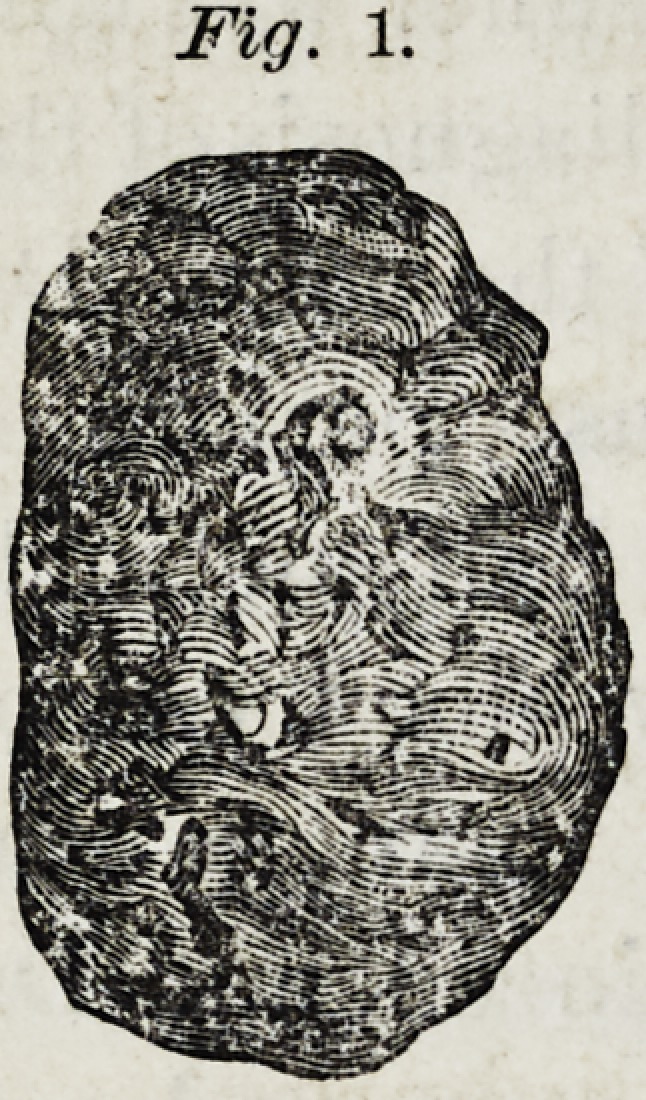


**Fig. 2. f2:**
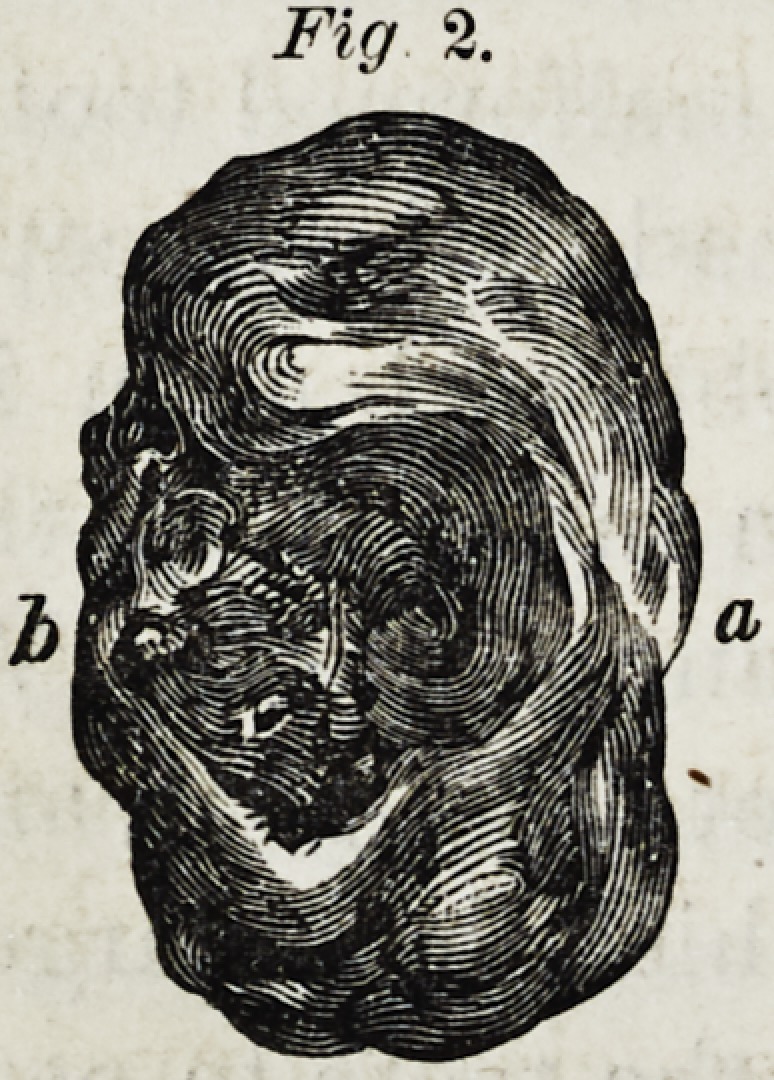


**Fig. 3. f3:**